# A Descriptive Study on the Extent of Dietary Information Obtained during Consultations at a Veterinary Teaching Hospital

**DOI:** 10.3390/ani12050661

**Published:** 2022-03-06

**Authors:** Andreina Schramm, Peter Hendrik Kook

**Affiliations:** Clinic for Small Animal Internal Medicine, Vetsuisse Faculty, University of Zurich, 8057 Zurich, Switzerland; andreina.schramm@uzh.ch

**Keywords:** food, history, response, owner, gastrointestinal, dog, referral

## Abstract

**Simple Summary:**

The majority of dogs with chronic idiopathic gastrointestinal (GI) disease are diet-responsive. We retrospectively evaluated what dietary information was gained when dogs were presented with chronic GI signs to either our Gastroenterology (GE) (*n* = 243) or Internal Medicine (IM) (*n* = 239) Service between 10/2017 and 01/2020. Referral documents mentioned the previously fed diet in 53/131 (40%) GE and 14/112 (13%) IM referrals. No dog had received more than one diet trial from the referring veterinarian. Diet trials had been performed in 127/199 (64%) GE and 56/156 (36%) IM dogs prior to presenting to our teaching hospital. The diet fed at the time of consultation could be named by 106/199 (53%) GE and 40/156 (26%) IM dog owners. Data on response to subsequent newly prescribed diets were available from 86 GE dogs and 88 IM dogs. A positive response to diet was noted in 50/86 (58%) GE and 26/88 (30%) IM dogs. A further 23/35 (66%) GE dogs and 12/21 (57%) IM dogs responded positively to a second diet trial, and 4/9 GE dogs (44%) and 6/7 (86%) IM dogs responded positively to a third diet trial. In conclusion, obtainable dietary information was infrequent. Positive response is still possible after initial diet failures. Further studies are now needed to determine if collecting complete dietary information at the time of consultations could lead to an increased percentage of diet-responsive disease.

**Abstract:**

The majority of dogs with chronic idiopathic gastrointestinal (GI) disease respond to diet. So far, no study has assessed how much dietary information is obtained during consultations. We retrospectively evaluated what dietary information was available from dogs presenting to our Gastroenterology (GE), and Internal Medicine (IM) Service between 10/2017 and 01/2020. Data from 243 dogs presenting for first GE consultations were compared to 239 dogs presenting with chronic GI signs for first IM consultations. Referrals comprised 131 (54%) GE dogs and 112 (47%) IM dogs. Referral documents specified the previously fed diet in 53/131 (40%) GE and 14/112 (13%) IM dogs. No dog had received more than one previous diet trial for chronic GI signs. Irrespective of referral status, diet trials had been performed in 127/199 (64%) GE, and 56/156 (36%) IM dogs. The specific diet fed at the time of consultation could only be named by 106/199 (53%) GE and 40/156 (26%) IM dog owners. Data on response to subsequent newly prescribed diets were available from 86 GE dogs and 88 IM dogs. A positive response to diet was noted in 50/86 (58%) GE and 26/88 (30%) IM dogs. A further 23/35 (66%) GE dogs and 12/21 (57%) IM dogs responded positively to a second diet trial, and 4/9 GE dogs (44%) and 6/7 (86%) IM dogs responded positively to a third diet trial. In conclusion, overall dietary information gained from referring veterinarians and owners was often incomplete. More dietary information could be gained during GE consultations compared to IM consultations for chronic GI signs. A positive response to diet can still be seen after two diet failures. Further studies will help to ascertain if the percentage of diet-responsive GI disease increases when more complete dietary information is obtained at the time of consultations.

## 1. Introduction

When assessing the response to therapy in dogs with chronic idiopathic gastrointestinal (GI) disease, food-responsive disease (FRD) has emerged as the most common entity, while antibiotic- and corticosteroid-responsive disease is less frequently documented [1]. This is best studied in dogs with chronic small intestinal enteropathy of varying severity [2,3,4,5,6,7], but also applies to dogs with chronic colitis [8], and dietary interventions are also successful when treating acute diarrhea [9,10]. Still, it is our long-term clinical experience that the majority of dog owners feeding commercial diets do not know what diet they have been feeding when asked during consultations. The situation is similar when assessing what dietary information is obtainable from referral documents. Not knowing the dog’s current and previous diets understandably complicates the choice of a new diet and thus can compromise dietary success. Similarly, the literature has not investigated treatment success after a failed diet trial in dogs with GI disease and it is our experience that costly and invasive diagnostics such as endoscopy are not immediately indicated after one failed diet trial.

We hypothesized that information on diet (what diet was fed and response to diet) would be scarce in referral documents, and that the majority of dog owners would not be able to name diets that had been tried before or were fed at the time of consultation. We further hypothesized that less dietary information would be obtained from owners presenting their dogs to the Internal Medicine (IM) Service compared to owners presenting their dogs to a Gastroenterology (GE) Service, a subspecialty of our IM Service. An ancillary aim was to investigate how many dogs did in fact respond positively to diet after a first diet trial had failed.

## 2. Materials and Methods

### 2.1. Case Selection and Data Collection

All data were obtained from the hospital information system Vetera^®^ (Vetera GmbH, 65344 Eltville am Rhein, Germany) at the Clinic for Small Animal Internal Medicine, Vetsuisse faculty, University of Zurich. Anamnestic information was recorded from dogs presented to the Gastroenterology (GE) Service, as well as to the general Internal Medicine (IM) Service for chronic GI signs.

Dogs presented for a first appointment between October 2017 and January 2020 were included. In order to be selected for the IM group, dogs had to present for chronic GI signs, and secondary GI disease (e.g., endocrine or renal disease) had to be ruled out. Because of the larger IM caseload, for each week the first 2 dogs presenting with chronic GI signs were selected for the IM group. Cases where it was apparent that a language barrier existed (for example when noted “owner speaks Russian, hardly any English”) were excluded.

### 2.2. Data Handling and Analysis

Demographic data (breed, age, sex, body weight, and referral status (yes/no)) were compiled. At our hospital, owners can also schedule an appointment for a second opinion without having been referred. All pretreatments (dietary and medical) for chronic GI signs were recorded. Pretreatment was subdivided into the following categories: dietary, probiotics, antibiotics, corticosteroids, or immunosuppressive drugs other than corticosteroids

Available information on previous diet trials and current diet as well as information from re-checks at our hospital were extracted from the clinic system. In referred patients, the referral documents were screened for information on previous diet trials (number of diet trials, name of diets) and response to diet. Our records were checked to see if the following questions were asked: what diet(s) had been fed (name), how many diets were tried before consultation, for how long had they been tried, and what were the responses. Whether the following information could be obtained from our records was also noted: the name of the dog’s current diet, if the diets were homemade or raw meat based (RMB), if the dog received any additional foods (e.g., cooked chicken, chicken hearts, cottage cheese) or other additives and supplements (e.g., vitamins, minerals, oils, fibers) or treats, and how many dogs received empirical dewormers.

It was further recorded if and what type of diet was prescribed by the attending clinician at our hospital. Diets were classified as highly-digestible (including low fat diets), hydrolyzed protein, or limited ingredient novel protein. Prescribed medical treatments were also recorded and categorized as treatment with probiotics, antibiotics, corticosteroids, and immunosuppressive drugs other than corticosteroids. If available, the same information was also recorded from first re-check consultations at our hospital.

The length of time the newly prescribed diet was fed and whether the response was positive was also recorded. Data on dietary compliance were recorded as compliant if the medical records mentioned that the owner was feeding the diet exclusively. Response to treatment was summarized as follows: 1. positive response to diet alone, 2. positive response to probiotics alone, 3. positive response to a combination of diet and probiotics, 4. positive response to antibiotics alone, or 5. positive response to corticosteroids alone.

Furthermore, medical files of all dogs were searched for information on additional diet trials performed at our hospital after the first re-check consultation. Length of diet trial and response was recorded. Due to the variation in the documentation of response to diet in our medical records, we summarized response to diet as either a “positive response to diet” or “negative response to diet”. A positive response was defined as any improvement in GI signs, whereas negative response was defined as either no response or a worsening of signs. Therefore, unfortunately, this could not be further subdivided, for example into full or partial response. Microsoft^®^ Excel (Version 16.35, for Mac) was used for data compilation, and calculation of median, ranges, and percentages.

## 3. Results

### 3.1. Study Population

A total of 243 dogs were included in the GE group and 239 dogs in the IM group. Demographic details can be found in Table 1. The most common breeds in the GE group were Crossbreeds (37/243, 15%), French Bulldogs (11/243, 5%), and Labrador Retrievers (10/243, 4%). While the most common dog breeds in the IM group were Crossbreeds (41/239, 17%), Poodles (13/239, 5%), and Labrador Retrievers (12/239, 5%).

### 3.2. Pretreatment

Information on pretreatment can be found in Table 2. Of those dogs that received probiotics, a high-dose (minimum of 200 billion bacteria) multi-strain probiotic [8] had been given to 7/23 (30%) of GE dogs and 2/25 (8%) of IM dogs. Treatment with empirical dewormers was recorded for 115 (47%) GE dogs, and 107 (45%) IM dogs.

### 3.3. Dietary Information Available from Referring Veterinarians

Table 3 outlines information gained from referral documents. All referred cases had chronic (3 or more weeks) GI signs from the time the referral was made. None of the referral documents mentioned more than one diet trial for the GI signs.

### 3.4. Dietary Information Gained during First Consultation

Table 4 outlines what dietary information could be gained during first consultations at our hospital. If more than one diet trial had been tried before consultation, no information on length of, or response to, those additional diet trials was available.

Only 122 of 199 (61%) GE dog owners and 54/156 (35%) of IM dog owners could name the manufacturer (e.g., Royal Canin) of the current diet. The specific diet (manufacturer and product) currently fed could only be named by 106/199 (53%) GE dog owners and 40/156 (26%) IM dog owners. Available information on what types of diet were fed can be seen in Table 5.

### 3.5. Homemade and Raw Meat-Based Diets

The distribution of homemade diets and raw meat-based diets among diets fed at the time of first consultation are presented in Table 6.

### 3.6. Dietary Additives and Treats

Additional foods and additives were fed to 35/199 (18%) dogs in the GE group and 33/156 (21%) dogs in the IM group, while 19/199 (10%) GE dogs and 13/156 (8%) IM dogs received treats. Receiving “no treats” was specifically recorded in 4/199 (2%) GE dogs and in 1/156 (<1%) IM dogs.

The three most frequently used additional foods and additives in the GE group were: cooked chicken 5/35 (14%), cottage cheese 4/35 (11%), and Canikur^®^ (Boehringer Ingelheim, Germany) (contains probiotics, clay, mannan-oligosaccharide) 2/35 (6%), and for the IM group: Cottage cheese 6/33 (18%), vitamins 3/33 (9%), and Bismutal^®^ (Graeub AG, Switzerland) (contains carob, kaolin) 2/33 (6%).

### 3.7. Dietary and Medical Therapy Prescribed during First Consultation

A new diet (i.e., diet change) was prescribed to 118/243 (49%) GE dogs and 153/239 (64%) IM dogs. The type of diets prescribed can be seen in Table 7.

The medical treatments prescribed during first consultations at our hospital are listed in Table 8.

### 3.8. Re-Check Consultations and Response to Diet and Medical Therapies

One hundred and fifty-nine of 243 (65%) GE dogs and 143/239 (60%) IM dogs were seen for a first re-check consultation after a median of 4 weeks (range 1–25 weeks) for GE dogs and 2 weeks (range 1–28 weeks) for IM dogs. Out of these, 92/159 (58%) GE dogs, and 89/143 (62%) IM dogs had received a new diet. The newly prescribed diet had been fed for a median of 3 weeks (range 1–24 weeks) in GE dogs and 2 weeks (range 1–22) in IM dogs.

Compliance to dietary and medical treatment was recorded for 60/92 (65%) GE dogs and 59/89 (66%) IM dogs. Compliance to dietary treatment alone was recorded for 9/12 (75%) GE dogs and 8/9 (89%) IM dogs.

Data on response to treatment were available for 143/159 (90%) GE dogs and 141/143 (99%) IM dogs. Data on response to new diet (and diet together with concurrent treatment) were available for 86/92 (93%) GE and 88/89 (99%) IM dogs and are presented in Table 9.

### 3.9. Positive Response to Second and Third Diet Trial

Thirty-five of 92 (38%) GE dogs and 21/59 (24%) IM dogs were prescribed a second diet. The second diet was fed for a median of 2 weeks in both groups (range GE 1–24 weeks; IM 1–22 weeks), 23 GE dogs (66%), and 12 IM dogs (57%) responded positively to the second diet. Data on a third diet trial were available in 9/35 (26%) GE dogs and 7/21 (33%) IM dogs. The third diet was fed for a median of 4 weeks (range 2–84) in GE dogs and 3 weeks (range 1–5 weeks) in IM dogs, 4 GE dogs (44%), and 6 IM dogs (86%) responded positively to the third diet. None of the dogs with a positive response to a second and third diet change received additional new treatments. Results can be seen in Table 10.

### 3.10. Change in Diet Type from First to Second Diet Trial

Twenty-six of 35 (74%) GE dogs and 13/21 (62%) IM dogs changed diet type between first and second diet trial. For GE dogs, the three main changes were from highly-digestible to hydrolyzed protein diets: *n* = 5 (19%), hydrolyzed protein to other diets: *n* = 5 (19%), and other diets to highly-digestible diets: *n* = 4 (15%). For IM dogs, the main changes were highly-digestible to hydrolyzed protein diets: *n* = 5 (38%), and highly-digestible to limited ingredient novel protein diets: *n* = 3 (23%).

### 3.11. Change in Diet Type from Second to Third Diet Trial

Six of nine (67%) GE dogs and five of seven (71%) IM dogs changed diet type between the second and third diet trial. For GE dogs, the main change was from hydrolyzed protein to other diets: *n* = 3 (50%).

## 4. Discussion

In this retrospective study, we investigated how much dietary information (e.g., exact types of diets fed, length of diet trials) can be obtained from referring veterinarians and dog owners when presenting to our hospital for chronic GI signs. The amount of dietary information collected during consultations has not yet been addressed in veterinary medicine. We conducted this study to highlight an apparent contradiction: On the one hand, it has become increasingly clear that the majority of dogs with chronic idiopathic GI diseases can be controlled by diet alone [2,3,6,7]; on the other hand—despite the well-established importance of diet—many owners still do not know what they are feeding when they present their dogs for consultations. It is also our experience that not only dog owners but also referring veterinarians seem to underestimate the impact of diet on their patients’ GI conditions.

At our institution, we offer a separate service within the Internal Medicine Service for patients presenting with GI problems. However, dogs with GI disease are also seen by the Internal Medicine Service as GE consultations are only available on 2 days per week compared to 5 days per week for IM consultations. This setting gave us the opportunity to compare dietary information taken at two services with different degrees of specialization and experience when treating dogs with GI disease. By doing so we could assess how results differ when dogs are seen by a larger group of veterinarians with differing interests in internal medicine versus a smaller service lead primarily by one internist focused on GE. We assumed that more dietary information would be retrievable from medical records if owners or referring veterinarians were seeking an appointment with the GE Service. Although our data confirmed this, diet was only mentioned in referral documents from 40% of cases referred to the GE Service and 13% referred to the IM Service. Referral documents including a diet history specification section might help to improve this. Unexpectedly, information on the actual response to previously tried diets was more frequently available in the IM group. However, this may have been due to selection bias as diet was overall only mentioned in 14/112 IM cases. We assume that one of the reasons for the low percentages of a specific mention of how dogs had responded to the previously tried diet, especially in the GE group, was that as the GI signs still persisted and necessitated a referral, a failed response was obvious and therefore mention of this was not warranted. The fact that none of the referral documents (GE and IM group) mentioned more than one previous diet trial may reflect the perception that failure to respond to one diet is synonymous with a general non-responsiveness to diet. Another possibility is that dog owners were not willing to pursue further diet trials, but rather wanted a second opinion or further diagnostic tests performed. In addition, dog owners might not perceive dietary treatment to be as effective as drug treatment in cases of GI disease. In order to better understand which factors motivate owners to try a diet for their dogs with GI signs, prospective survey studies are needed.

More owners in the GE group were asked about their dog’s diet (82 vs. 65% IM group). Therefore, 18% of owners coming in for a GE consultation were not asked about their dog’s diet. It is possible that nothing was recorded in cases where owners did not know what they were feeding. Alternatively, lacking data here may have been due to negligent documentation. Irrespective of referral status, approximately two-thirds in the GE group and only one-third of dogs with chronic GI signs seen by the IM Service had a previous diet trial recorded, and clearly more information on response to previous diet trials were recorded in the GE group (86%) vs. IM group (45%). Again, not recording response rates may have been synonymous with the tacit assumption that GI signs did not improve with the mentioned diet.

The most impressive result of this study is that only 106/199 (53%) dog owners seeking medical advice from the GE Service could in fact name the specific diet their dog was receiving at the time of consultation. Even worse, only 40/156 (26%) owners in the IM group knew the specific diet currently fed to their dog. Frequently, owners assumed they knew what they were feeding when they could only recall the manufacturer of the diet (e.g., Royal Canin); not realizing that one manufacturer can produce a multitude of different diets. It is not uncommon for the last author to sit together with dog owners in front of the computer screen searching for images of what their dog’s food bag looks like in an attempt to identify the current diet—knowing so little about previously fed diets is probably one reason for the frequent selection of a highly digestible or hydrolyzed protein diet for the first diet trial, as more specific aspects such as dietary protein remain often unknown.

Approximately 20% of all dogs were fed additional foods besides their diet, and 10% received treats. These numbers are higher than what have been reported for healthy dogs from the U.S. and Australia [11]. This is likely because owners of sick dogs are keen to ensure that their dog consumes at least some food. It is possible that our results are still an underestimation, as we cannot guarantee with a retrospective study design that these questions were routinely asked, especially as treats are fed more often according to other studies [11,12]. Additional foods or treats besides the prescribed diet may be responsible for diet failure. However, only very few studies on chronic enteropathies in dogs have excluded treats in their study design [13,14,15]. The situation is similar when assessing studies on acute diarrhea in dogs [9].

Considerably more dogs in the GE group received high-dose multi-strain probiotics often together with a new diet compared to the IM group, which is likely related to an increased awareness of the benefits of probiotics in the GE Service. The combination of diet with a probiotic makes it difficult to assess which treatment the dog ultimately responded to. Explicit mention of exclusive positive response to probiotics was noted in only a handful of cases. Further differentiation between diet and probiotic responsiveness might be interesting for future studies on therapeutic outcome, as a recent study showed clinical, histological, and immunomodulatory benefits of a high-dose multi-strain probiotic in dogs with inflammatory bowel disease when all dogs remained on their pre-trial diets [16].

Approximately 150 dogs (=60%) were seen for a first re-check in both groups. The reason for longer time intervals between first consultation and re-check between groups was that the GE Service offers less consultation appointments compared to the IM Service. Compliance to diet, either as the sole treatment or more commonly in combination with other treatments, was recorded in two-thirds of cases in both groups (GE 69/104 (66%), IM 67/98 (68%)). This is comparable to dietary compliance reported in dogs with atopic skin disease (73% dietary compliance) [17]. Similar dietary compliance (69.6%) of owners was shown in a study on homemade diets in dogs [18]. Interestingly, no data on compliance to therapy could be recorded during later re-checks. Our compliance data imply that one-third of dogs were either not fed the newly prescribed diet exclusively or were not given medication as prescribed. However, it is also possible that clinicians were not asking and recording compliance data. Dogs treated with diet only, had a higher compliance (GE 75%, IM 89%); most probably as these dogs had milder disease and thus less inappetence. Understandably, lacking compliance can affect response rates to diet. So far, this factor has not been specifically considered in studies on dogs with GI disease. Dietary compliance is discussed as a key point of success for the therapy of chronic enteropathies [1,6,7,19,20], but specific documentation of dietary compliance is found very rarely in the literature [4,21,22]. Prospective studies are needed for a more precise characterization of dietary compliance.

When assessing all available data from all dogs receiving a new diet, including dogs that were additionally treated with probiotics, a positive response to diet was recorded in 50/86 (58%) GE dogs and 26/88 (30%) IM dogs. These numbers might be higher, had more owners known what they were feeding at first consultation because two-thirds of 35 dogs in the GE group still responded positively to a second diet trial after failing the first diet. However, additional studies would be needed to assess this. A positive response to a stringent diet after referral despite previous unsuccessful diet trials has been reported [13], but it is usually not specified in the literature how many diets were tried before dogs were designated as FRD or not. It is conceivable that this is one of the reasons why none of the referral letters contained information on more than one previous diet trial.

A second diet was tried less commonly in the IM group, which may have been due to different awareness of the importance of diet. When planning the study, we had expected more positive responses to a third diet change. We believe the comparably small data set on responses to a third diet trial was due to the fact that only consultations at the hospital and no telephone updates were included. However, when taking both groups into consideration, 10/16 (63%) dogs still responded to diet after having failed two previous diet trials.

When taking into account all positive responses to all three diet trials, then 77/86 (90%) GE dogs, and 44/88 (50%) IM dogs responded positively to diet. These figures must be viewed with caution, as a standardized and graduated assessment of response to diet was not available. In addition, numbers depend on the underlying disease process and how convincingly the relevance of diet has been communicated to owners in both services. The difference between services is certainly also influenced by the low rate of identified diets at first consultation in the IM group (26%).

The variability of diet trial duration in our study might have been due to owner specifics (i.e., differing patience or expectations regarding dietary success), differing recommendations of attending clinicians, and also in part due to limited availability of follow-up appointments.

The limitations of our study are the typical limitations of a retrospective study design. Whenever data were not available, we did not know if this was on behalf of the attending clinician not asking all required questions (e.g., compliance), negligent documentation, or owner unable to recall the specific details of the diet fed. Similar to large-scale studies on long-term outcome in dogs with CE [2], we graded the response to diet subjectively (improved versus not improved) and did not document specifically which clinical signs had improved and by how much. Our primary goals were to investigate how much dietary information could be gained from referring veterinarians and from owners of dogs with chronic idiopathic GI signs, and also how many dogs still improve clinically with diet after a failed diet trial. A full diagnostic work-up including endoscopy was not a prerequisite of this study. Therefore, results may differ when related to a more defined disease process in future studies, as diet can play a main role in the therapy or represent a supportive measure depending on the diagnosis. It would be interesting to compare our results to other institutions, but this is the first study evaluating the extent of obtainable dietary information during a veterinary consultation.

## 5. Conclusions

Dietary information gained from referring veterinarians and owners of dogs presented for chronic GI signs is often incomplete or lacking. Dietary information appeared more often complete when dogs were presented to the GE Service, but this was still just over half of the cases. A substantial percentage of dogs still improve clinically after having failed a first diet trial. Further studies will help to ascertain if the percentage of diet-responsive GI disease increases when more complete dietary information is obtained at the time of consultations.

## Figures and Tables

**Table 1:** Demographic details on study population (GE = Gastroenterology, IM = Internal Medicine).

	GE Dogs (*n* = 243)	IM Dogs (*n* = 239)
Age (years), Median (range)	8 (1–19)	8 (1–18)
Sex	133/243 (55%) males	131/239 (55%) males
	72/133 (54%) neutered	69/131 (53%) neutered
	109/243 (45%) females	108/239 (45%) females
	78/109 (72%) spayed	74/108 (69%) spayed
Body weight (kg), median (range)	11.1 (0.9–61)	13.6 (1.3–62.9)

**Table 2:** Pretreatment, summarized into categories (GE = Gastroenterology, IM = Internal Medicine).

	GE Dogs (*n* = 243)	IM Dogs (*n* = 239)
Probiotics	23/243 (9%)	25/239 (10%)
Corticosteroids	35/243 (14%)	26/239 (11%)
Other immunosuppressive drugs	5/243 (2%)	3/239 (1%)
Antibiotics	50/243 (21%)	49/239 (21%)

**Table 3:** Diet-related information available from referring veterinarians for dogs presenting with chronic GI signs (GE = Gastroenterology, IM = Internal Medicine).

	GE Dogs	IM Dogs
Number of referred dogs	131/243 (54%)	112/239 (47%)
Previous diet mentioned in referral	53/131 (40%)	14/112 (13%)
Response to diet mentioned in referral	16/53 (30%)	9/14 (64%)

**Table 4:** Dietary information gained from owners of dogs with chronic GI signs during first consultation at our hospital (GE = Gastroenterology, IM = Internal Medicine).

	GE Dogs	IM Dogs
Clinician inquired on dog’s diet	199/243 (82%)	156/239 (65%)
Previous diet trial performed	127/199 (64%)	56/156 (36%)
No. of previous diet trials (med., range)	1 (1–5)	1 (1–6)
Length of previous diet trial (med., range)	4 weeks (1–25)	2 weeks (1–8)
Response to previous diet trial recorded	109/127 (86%)	25/56 (45%)
Only manufacturer of diet could be named for current diet	122/199 (61%)	54/156 (35%)
Specific diet (manufacturer and product) currently fed named	106/199 (53%)	40/156 (26%)

**Table 5:** Type of diets fed at the time of first consultation at our hospital (GE = Gastroenterology, IM = Internal Medicine).

Type of Diet	GE Dogs *n* = 106	IM Dogs *n* = 40
Highly-digestible diet	40 (38%)	4 (10%)
Hydrolyzed protein diet	21 (20%)	1 (3%)
Limited ingredient novel protein diet	7 (7%)	1 (3%)
Other diet	37 (35%)	7 (18%)

**Table 6:** Homemade diets and raw meat-based diets (RMBD) fed at the time of first consultation to our hospital (GE = Gastroenterology, IM = Internal Medicine).

Type of Diet	GE Dogs *n* = 199	IM Dogs *n* = 156
Homemade diet	57/199 (29%)	31/156 (20%)
RMBD	24/199 (12%)	10/156 (6%)

**Table 7:** Type of diets prescribed at first consultation at our hospital. Highly digestible diets included Royal Canin Veterinary Diet Sensitivity Control, Hill’s Prescription Diet i/d, Hill’s Prescription Diet i/d Sensitive, Hill’s Prescription Diet i/d Low Fat. Hydrolyzed protein diets included Royal Canin Veterinary Diet Anallergenic, Hill’s Prescription Diet z/d, Purina Pro Plan Veterinary Diets HA. Limited ingredient novel protein diets included Essendia Exclusion limited ingredient novel protein diets, Vet-concept limited ingredient novel protein (Sana) diets, Hill’s Prescription Diet d/d. “Other diet” refers to any other diet. (GE = Gastroenterology, IM = Internal Medicine).

Type of Diet	GE Dogs *n* = 118	IM Dogs *n* = 153
Highly-digestible (incl. low-fat) diet	46 (39%)	95 (62%)
Hydrolyzed protein diet	36 (31%)	23 (15%)
Limited ingredient novel protein diet	17 (14%)	7 (5%)
Other diet	19 (16%)	28 (18%)

**Table 8:** Medical treatment prescribed at first consultation at our hospital. All high-dose multi-strain probiotics were SivoMixx^®^ (Ormendes SA, 1008 Jouxtens-Mézery, Switzerland) (GE = Gastroenterology, IM = Internal Medicine).

Medical Treatment	GE Dogs *n* = 172	IM Dogs *n* = 224
High-dose multi-strain probiotics	105/172 (61%)	63/224 (28%)
Antibiotics	13/172 (8%)	45/224 (20%)
Corticosteroids	24/172 (14%)	19/224 (8%)
Other immunosuppressive drugs	6/17 (3%)	0 (0%)

**Table 9:** Available data on positive response to diet change at first re-check at our hospital (GE = Gastroenterology, IM = Internal Medicine).

Positive Response to Treatment	GE Dogs (*n* = 86)	IM Dogs (*n* = 88)
Only diet change	19/86 (22%)	21/88 (24%)
Diet change with probiotics	31/86 (36%)	5/88 (6%)
Diet change with corticosteroids	0/86 (0%)	4/88 (5%)
Diet change with antibiotics	0/86 (0%)	6/88 (7%)

**Table 10:** Available responses to second and third diet change documented at our hospital. A positive response was defined as any improvement in GI signs, whereas a negative response was defined as either no response or a worsening of signs. (GE = Gastroenterology, IM = Internal Medicine).

**Outcome after Second Diet Change**	**GE Dogs (*n* = 35)**	**IM Dogs (*n* = 21)**
Positive response	23/35 (66%)	12/21 (57%)
Negative response	7/35 (20%)	4/21 (19%)
No data	5/35 (14%)	5/21 (24%)
**Outcome after Third Diet Change**	**GE Dogs (*n* = 9)**	**IM Dogs (*n* = 7)**
Positive response	4/9 (44%)	6/7 (86%)
Negative response	1/9 (11%)	0/7 (0%)
No data	4/9 (44%)	1(7 (14%)

## Data Availability

Data are only available on request due to restrictions, e.g., privacy or ethical reasons. As the study is retrospective, no owner consent was signed and personal data are therefore not publicly available.
